# HR LC-MS/MS metabolomic profiling of *Yucca aloifolia* fruit and the potential neuroprotective effect on rotenone-induced Parkinson’s disease in rats

**DOI:** 10.1371/journal.pone.0282246

**Published:** 2023-02-28

**Authors:** Dalia E. Ali, Samar M. Bassam, Soha Elatrebi, Esraa S. Habiba, Eman A. Allam, Eman M. Omar, Doaa A. Ghareeb, Shaymaa A. Abdulmalek, Essam Abdel-Sattar

**Affiliations:** 1 Pharmacognosy and Natural Products Department, Faculty of Pharmacy, Pharos University, Alexandria, Egypt; 2 Clinical Pharmacology Department, Faculty of Medicine, Alexandria University, Alexandria, Egypt; 3 Medical Physiology Department, Faculty of Medicine, Alexandria University, Alexandria, Egypt; 4 Biochemistry Department, Faculty of Science, Alexandria University, Alexandria, Egypt; 5 Pharmacognosy Department, Faculty of Pharmacy, Cairo University, Giza, Egypt; Foshan University, CHINA

## Abstract

*Yucca aloifolia* L. fruit (*Yucca* or Spanish bayonet, family Asparagaceae) is recognized for its purplish red color reflecting its anthocyanin content, which has a powerful antioxidant activity. This study aimed to investigate yucca (YA) fruit extract’s protective effect on Parkinson’s disease (PD). *In vitro* study, the anti-inflammatory activity of yucca fruit extracts was explored by measuring tumor necrosis factor receptor 2 (TNF-R2) and nuclear factor kappa B (NF-_*K*_B) to choose the most effective extract. Afterward, a detailed *in vivo* investigation of the protective effect of the most active extract on rotenone-induced PD was performed on male albino Wister rats. First, the safety of the extract in two different doses (50 and 100 mg/kg in 0.9% saline orally) was confirmed by a toxicological study. The rats were divided into four groups: 1) normal control (NC); 2) rotenone group; and third and fourth groups received 50 and 100 mg/kg yucca extract, respectively. The neurobehavioral and locomotor activities of the rats were tested by rotarod, open field, and forced swim tests. Striatal dopamine, renal and liver functions, and oxidative stress markers were assessed. Western blot analysis of brain tissue samples was performed for p-AMPK, Wnt3a, and β-catenin. Histopathological examination of striatal tissue samples was performed by light and electron microscopy (EM). The metabolites of the active extract were characterized using high-resolution LC-MS/MS, and the results showed the prevalence of anthocyanins, saponins, phenolics, and choline. Biochemical and histopathological tests revealed a dose-dependent improvement with oral *Yucca* extract. The current study suggests a possible neuroprotective effect of the acidified 50% ethanol extract (YA-C) of the edible *Yucca* fruit, making it a promising therapeutic target for PD.

## Introduction

Parkinson’s disease (PD) is the second most common neurological disease after Alzheimer’s. It is characterized by motor dysfunction in the form of stiffness, slowness, resting tremors, poor posture reflexes, and non-motor dysfunction as mood abnormalities, cognition problems, sleep changes, and impaired autonomic function [1]. The main pathological feature of PD is the degeneration of dopaminergic neurons in the substantia nigra pars compacta (SNpc). The etiology of PD is unknown, but factors such as mitochondrial dysfunction, oxidative stress, neuroinflammation, toxic factors, and genomic factors are associated with it [2].

Currently, there is no definitively effective cure for PD, despite the availability of many pharmacotherapeutic agents that slow the disease’s fatal progression. These have many side effects and limitations and usually do not target an exact mechanistic pathway. Consequently, dietary elements can help control clinical deterioration with a good safety profile [3]. Anthocyanins and their metabolites are a group of dietary water-soluble pigments with anti-oxidative and anti-neuroinflammatory activities tested in several animal models of neurological disorders [1, 3].

It has been found that upregulation of Wnt/β-catenin signaling enhances dopaminergic neurogenesis by regulating proneural genes (Nurr-1, nuclear receptor related 1 protein, Pitx-3, pituitary homeobox 3 protein, Ngn-2, neurogenin 2 protein, and NeuroD1, neurogenic differentiation 1 protein) and mitochondrial biogenesis in SNpc in parkinsonian rats [4]. Additionally, the Wnt/β-catenin signaling pathway regulates the differentiation of mesenchymal stem cells through improving β-catenin/T-cell transcription factor 1 (TCF) mediated transcription. AMP-activated protein kinase (AMPK) phosphorylates β-catenin at Ser 552, stabilizing β-catenin and increasing β-catenin/TCF-mediated transcription [5]. Anthocyanins were shown to modulate AMPK, thus regulating the AMPK/Wnt pathway [6, 7]. Therefore, pharmacological manipulation of Wnt/β-catenin/AMPK signaling with anthocyanins-rich extracts could enhance the endogenous regenerative capacity of dopaminergic neurons, which would positively impact the course of the disease.

The phytochemical analysis using GC-MS of the saponifiable fraction of *Y*. *aloifolia variegata* cultivated in Egypt has resulted in the identification of 26 fatty acids, with palmitic acid and palmitoleic acid representing the major saturated fatty acids. At the same time, γ-sitosterol was the major compound of the unsaponifiable fraction [8]. The same authors analyzed the leaf extract of *Y*. *aloifolia variegata* using LC–ESI–MS/MS in negative and positive-ion modes, allowing the tentative identification of 41 and 34 compounds, respectively. This resulted in the identification of ten spirostanol saponin glycosides, in addition to phenolic acid derivatives (gallic, caffeic acids, and caffeic acid aminoglycoside), flavonoids (hesperidin, rutin, luteolin, acacetin, kaempferol, quercetin, and apigenin), stilbene derivatives, and a coumarin [8]. Mokbli et al. studied the seed oil extracted from *Y*. *aloifolia* seeds [9]. The principal fatty acids in the oil were linoleic acid, oleic acid, and palmitic acid, in addition to the high content of vitamin E, particularly tocotrienols. The pericarp of *Y*. *gloriosa* fruit afforded eight spiro- and furostanol glycosides of tigogenin derivatives [10].

Yucca had known ethnobotanical significance for Native Americans. Yucca is used mainly for its inflammatory conditions and is efficient in treating joint pain, bleeding, and urethral and prostate inflammations [11]. Yucca products marketed as supplements for joint pain depend mainly on their anti-inflammatory reputation [12]. *Yucca aloifolia* is an intensely colored, reddish-purple fruit expected to contain a high concentration of anthocyanins and other anti-inflammatory metabolites reported in other organs [13]. Yucca extracts’ anthocyanins and other metabolites are expected to improve PD [1, 13]. According to the authors, this is the first report on *Y*. *aloifolia* extract chemical profiling and its effect on PD. Therefore, it is interesting to investigate the possible protective effect of yucca fruit extract on rotenone-induced PD-like neurotoxicity and its possible mechanism of action.

## Materials and methods

### Plant materials

*Yucca aloifolia* L. fruits (Syn. *Y*. *aloifolia* L. var. aloifolia) were collected from El-Montaza Palace Garden, Alexandria, Egypt, in November 2020 (31°17′19′′N 30°00′57′′E; https://goo.gl/maps/CJo7eSqkKKrKuN5H8). The sample was identified by Mrs. Therese Labib, a botanical specialist and consultant at Orman and Qubba Botanic Gardens. The plant name was verified with the plant list (http://www.theplantlist.org/). Voucher samples of the plant (2020-2-25A) were preserved at the Faculty of Pharmacy, Cairo University (El-Kasr El-Aini Street, 11562 Cairo, Egypt; https://goo.gl/maps/3hwVQk6zJEpBCaEP8).

### Preparation of the plant extracts

Four extracts were prepared from fresh fruit (30 g each) to select the extract with the highest phenolic content. The plant material was separately extracted (for 12 h at dark) with 100 mL of acidified (0.1% TFA) ethanol (YA-A), acidified 80% ethanol (YA-B), acidified 50% ethanol (YA-C), and acidified distilled water (YA-D). Each extract was filtered and concentrated under reduced pressure at 30°C and then freeze-dried and kept at 4°C till used.

### Preparation of anthocyanin rich-fraction

The anthocyanin fraction (YA-E) was prepared the same way as that reported by Cahlíková L. et al. [14]. Fresh fruit (30 g) was extracted twice at room temperature with a 10-fold volume of 95% ethanol (w/v) for 24 h, with continuous stirring. The ethanolic extracts were collected and evaporated under reduced pressure (30°C) to about 50 mL, and then a 10-fold volume of diethyl ether was added. After 2 h of standing, the liquid was decanted, and the reddish brown precipitate was dried in a desiccator for 24 h in the dark. This sediment was dissolved in 95% ethanol at room temperature, and a further 10-fold volume of diethyl ether was added. The liquid was poured, and the precipitate was dried in a desiccator for 24 h in the dark to obtain a dried deep reddish brown powder (0.36 g).

### Chemical characterization of the extracts

#### Determination of the total phenolic content

Total phenolics were determined employing the Folin–Ciocalteu method as previously described [15].

#### Determination of the total flavonoid content

Total flavonoid content was measured by the aluminum chloride colorimetric assay described by Kirnanmai et al. [16].

#### High-resolution LC-MS/MS metabolic profiling of *Y*. *aloifolia* fruit extract

Analysis was performed on a Triple TOF 5600+ nano LC-HRMS/MS device. HPLC separation was performed on (Waters) Exion Xbridge C18 column (2.1×50 mm, 3.5 μm) preceded by a (Phenomenex) pre-column, with in-Line filter disks (0.5 μm×3.0 mm). The mobile phase consisted of two solvents for each mode: solvent (A) deionized water containing 0.1% formic acid, solvent (B) a 5 mM ammonium formate buffer (pH 8) containing 1% methyl alcohol, and solvent (C) 100% acetonitrile. For the negative mode, (A) and (C) were used, while for the positive mode, (B) and (C) were used. A 20 μl stock (50/1000 μl) was diluted with 1000 μl reconstitution solvent. The injected concentration was 1 μg/μl. Gradient elution analysis was carried out at a rate of 0.3 mL/min at 40°C, where from 0 to 1 min, isocratic (90% (A) or (B), 10% (C)), from 1 to 25 min, linear gradient from 90 to 10% (A) or (B), 10% to 90% (C). From 25.01 to 28 min, elution was isocratic (90% (A) or (B), 10% (C)). Solvent (A) was used for negative ion mode only, while solvent (B) was used for positive ion mode. Conditions for MS ionization in the negative ion mode: the run duration was 28 min, including 2584 cycles, 0.6502 sec each. The range of mass detected was from 50 to 1000 Da. For the MS1 acquisition, nebulizer gas GS1 (nitrogen), drying gas GS2 (nitrogen), and curtain gas CUS flow rates were 45, 45, and 25 psi, respectively. The temperature was 500°C, and the ion spray voltage was −/+ 4500 V according to the mode. For MS2 acquisition, a declustering potential of 80 V, collision energy CE of 35 V, and collision energy spread CES of 20 V were applied, respectively. The maximum number of candidate ions to monitor per cycle was 15.

### *In vitro* biological study

#### Anti-inflammatory screening

Tumor necrosis factor receptor 2 (TNF-R2) and nuclear factor-kappa B (NF-_*K*_B) were measured by ELISA kit (# 79756, BPS Bioscience, USA, and #CSB-EL015761HU, Cusabio, China, respectively), according to the manufacturer’s instructions. Briefly, using a 96-well plate, the appropriate antibodies included in the kit and the extracts were allowed to react, followed by recording absorbance values that were assessed colorimetrically using a microplate reader [17]. All experiments were performed in triplicates. The extract(s) with promising *in vitro* anti-inflammatory effects were chosen for further *in vivo* study.

### *In vivo* biological study

#### Animals

The current study was conducted on 56 male albino Wistar rats weighing 150–190 g (6 weeks old). Rats were obtained from the El-Mouwasat animal house at the Alexandria Faculty of Medicine, Egypt, and kept in a standard laboratory environment with a temperature of 22°C and a 12-h light/12-h dark cycle. All animals were given free access to food and water and left for acclimatization before the study. The experimental protocol was approved by the Research Ethics Committee of the Alexandria Faculty of Medicine, Egypt (IRB code 00012098-FWA: No. 00018699; membership through Alexandria University in the International Council of Laboratory Animal Science organization, ICLAS).

#### Toxicological study

An initial toxicological study [3] was performed to confirm the safety of the most active *Y*. *aloifolia* extract on the liver, kidney, and dopaminergic neurons. Rats were randomly divided into three groups, each consisting of 8 rats. The first normal control (NC) group received 2 mL/kg of oral 0.9% saline daily for four weeks. The second and third groups received 50, and 100 mg/kg of oral *Yucca* extract dissolved in 0.9% saline daily for four weeks. One day after the last dose, behavioral tests were conducted, then they were sacrificed, and both striata were dissected. The isolated right striata were stored at -80°C for biochemical analysis of dopamine (DA) content. The other one was fixed in 10% formalin for further histological examination. Moreover, serum samples were used to assess liver enzymes and renal function.

#### Experimental design

After confirmation of the safety of *Y*. *aloifolia* extract, rats were randomly divided into four groups as follows (n = 8 per group): group 1; NC group: received 2 mL/kg 0.9% saline by oral gavage followed 20 min later by 1 mL/kg 2.5% DMSO (Sigma-Aldrich, MO, USA), subcutaneous (s.c.) for four weeks; group 2: rotenone group received 2 mL/kg 0.9% saline by oral gavage followed 20 min later by rotenone (Sigma-Aldrich, St. Louis, MO, USA) suspended in 2.5% DMSO (1.5 mg/kg/day, s.c.) for four weeks; group 3 and 4: received active *Yucca* extract (50 and 100 mg/kg, respectively) dissolved in 0.9% saline by oral gavage, then 20 min later, they received rotenone (as the second group) for four weeks.

Twenty-four hours following the last dose, rats were tested for neurobehavioral and locomotor abnormalities in addition to depression. They were sacrificed after being anesthetized with ketamine hydrochloride (30 mg/kg IM) and xylazine hydrochloride (5 mg/kg IM). Then blood was collected from the abdominal aorta, and their brains were dissected. The isolated striata from one hemisphere were stored at -80°C until further processing for biochemical analysis. The second striata were fixed in either 10% neutral formalin or 2.5% glutaraldehyde for subsequent light and electron microscopic histopathological examination.

### Neurobehavioral and locomotor tests

#### Rotarod test

The rotarod test was used to assess motor coordination and balance. All rats underwent a 3-day training program, after which a steady baseline level of performance was achieved. During this time, the rats were trained to walk against the motion of a rotating drum at a constant speed of 12 rotations per minute (RPM) for a maximum of 2 min. Four training attempts were conducted daily, with one-hour intervals between attempts. The rats that fell during the training attempt were placed back on the rotating drum. After the training, a one-day test of three trials was performed using the apparatus’s accelerating speed mode (4 to 40 RPM) for 5 min. The latency to fall off the Rotarod was recorded, and the mean of the three trials was calculated for each rat. Rats that stayed on the drum for 300 s without falling were removed, and their time was scored as 300 s. The apparatus was cleaned with 70% ethanol and dried before each trial [18].

#### Open field test

A wooden, rectangular, light brown-colored open-field apparatus of 50 x 30 x 20 cm was used to assess spontaneous locomotor and rearing activity. The apparatus floor was divided into 25 rectangular squares. The rat was placed on a corner square of an open-field apparatus facing the wall, and the number of squares crossed during the last 3 min was recorded. Each crossing was considered only when all four paws were in another square. The number of rears and latencies to move and to the rear were recorded. Following each trial, the apparatus was thoroughly cleaned [19].

#### Forced stress swim test

A forced stress swim test was used to assess depression. The rodents were exposed to a short, acute period of stress, recording the time and representing their response in an active rather than a passive way [20]. The test was performed over two days; the first day was a training day, in which the rats were placed in a tank of water (20 x 20 x 40 cm) at a temperature of 24 ± 1°C for 15 min at a depth of 15 cm. The next day they were subjected to a 5-min forced swim test, which was videotaped for the quantification of the following parameters: immobility time (defined as the lack of motion of the entire body, consisting only of the small movements necessary to keep the rat’s head above the water); climbing time (vigorous movements with forepaws in and out of the water, usually directed against the tank wall); and swimming time (was considered when large forepaw movements displacing the body around the cylinder, more than necessary to maintain the head above the water surface). To minimize the effects of material and temperature, the water was replaced after each rat.

### Estimation of biochemical markers

#### Determination of renal function and liver enzymes

Serum was obtained by centrifuging the collected blood at 1000×g for 15 min. It was used for the enzymatic analysis of urea, creatinine, serum glutamic-oxaloacetic transaminase (SGOT), and serum glutamic-pyruvic transaminase (SGPT) using the Hitachi 911 chemical analyzer (Roche Diagnostics, GmbH, Mannheim, Germany) according to the manufacturer’s instructions.

#### HPLC assay of striatal dopamine level

Deproteination and homogenization of striatal brain samples were performed using 0.2 M perchloric acid containing 100 M EDTA2Na. Samples were maintained for 30 minutes before being centrifuged at 10,000 g for 15 minutes to deproteinize them. The pH of the supernatant was adjusted to 3.35 after centrifugation using 1 M acetic acid. The supernatant was used for the determination of DA by HPLC. A total of 20 μl of supernatant containing paracetamol (100 mg/mL) as the internal standard was isocratically eluted through a C18 column (4.6 x 250 mm, 5 μm), with a mobile phase containing 50 mM ammonium phosphate pH 4.6, 25 mM hexane sulfonic acid pH 4.04, and 5% acetonitrile, and detected by a UV detector at 254 nm. The flow rate was 1 mL/min. An external standard was used to calibrate the data. The amount of DA was measured in micrograms in each mg of protein.

#### Lipid peroxidation assay (Methylenedioxyamphetamine detection)

The procedure for determining lipid peroxidation in rat brain homogenate was the same as previously described [21]. Methylenedioxyamphetamine (MDA) is a lipid peroxidation end product that combines with thiobarbituric acid to create a reactive molecule of pink chromogen–thiobarbituric acid. A polytron homogenizer was used to homogenize rat brains in 20 mM Tris-HCl, pH 7.4 (10 mL), at 4°C. The supernatant was recovered after centrifuging the homogenate at 1000 g for 10 min at 4°C. Then, 0.1 mL of the supernatant was mixed with 1.5 mL of acetic acid (15%), 1.5 mL of thiobarbituric acid (0.8%), and 0.2 mL of sodium dodecyl sulfate (8.1%) and heated at 100°C for 60 min. After cooling the mixture, 5 mL of n-butanol-pyridine (15:1) was added, along with 1 mL of distilled water, and vortexed rapidly. The organic layer was separated after centrifugation at 1200 g for 10 min, and the absorbance was measured at 532 nm using an ELISA plate reader.

#### Assessment of Nitric oxide (NO)

The amount of nitric oxide (NO) in the sample was determined using the Griess reaction [22], which involved mixing 100 μl of the sample with 100 μl of acidic Griess reagent (1% sulfanilamide and 0.1% naphthyl ethylenediamine dihydrochloride in 2.5% phosphoric acid). The NO level was calculated using the following equation:

$$\text{NO}\;\text{level}\;\left(\text{M}\right)=\text{Absorbance}\;\text{of}\;\text{test}/\text{Absorbance}\;\text{of}\;\text{standard}\;\text{times}\;\text{concentration}\;\text{of}\;\text{standard},\;\text{represented}\;\text{as}\;\mu \text{M}/\text{mg}\;\text{protein}.$$

#### Measurement of glutathione (GSH)

The previously reported method was used to measure GSH [23]. 100 μl samples, distilled water, and GSH were mixed with 100 μl sulphosalicylic acid (4%) and maintained at 4°C for at least 1 h before centrifugation at 1200 g for 10 min at 4°C. After that, 100 μl of the supernatant was combined with 2.7 mL of phosphate buffer (0.1 M, pH 7.4) and 0.2 mL of DTNB (5,5’-dithiobis-2-nitrobenzoic acid) and incubated for 5 min. The generated yellow color was measured spectrophotometrically at 412 nm, and a standard curve was created using standard GSH. Finally, the GSH content was measured in μg/mg protein.

#### Estimation of superoxide dismutase (SOD)

The approach outlined earlier by Raja et al. was used to assess the SOD activity [24]. The supernatant (0.1 mL), 1.2 mL of sodium pyrophosphate buffer (pH 8.3; 0.052 M), 0.3 mL nitro blue tetrazolium (300 μm), 0.1 mL of phenazine methosulphate (186 μm), and 0.2 mL NADH (750 μm) were included in the assay mixture. NADH was used to start the reaction, and the reaction was stopped by adding 0.1 mL of glacial acetic acid after 90 s of incubation at 30°C. Then 4.0 mL of n-butanol was added, and the reaction mixture was rapidly agitated. The chromogen’s color intensity in n-butanol was determined spectrophotometrically at 560 nm, and SOD content was expressed as U/mg protein.

#### Western blot analysis

The brain tissues were homogenized on ice in radioimmunoprecipitation (RIPA) buffer and centrifuged at 10,000 rpm for 30 min to separate the supernatant for western blot analysis. A bicinchoninic acid (BCA) protein assay was used to assess protein content. The samples were performed on 12.5% SDS-PAGE gels with a total protein loading volume of 40 μg per lane. A semi-dry transfer technique was used to transfer the isolated proteins to a PVDF membrane (Bio-Rad, Hercules, CA, USA). After blocking with 5% nonfat milk in TBST, the membranes were incubated overnight at 4°C with the primary antibodies of p-AMPK (50081), Wnt3a (2721), β-catenin (8480), and β-actin (4970). Membranes were washed with Tris-buffered saline with Tween (TBST) following primary antibody incubation and treated with anti-rabbit (1:1000) secondary antibody, after which they were rinsed with TBST. The image was analyzed with Image Lab software (Bio-Rad Laboratories, Hercules, CA, USA). The experiment was repeated three times, and the intensity of each band was adjusted against β-actin levels.

#### Estimation of total protein

Protein levels were calculated using Lowry’s method from rat striatal brain sections that had previously been utilized for DA assays and antioxidant enzyme calculations [25].

### Histopathological examination

#### Striatal hematoxylin and eosin staining

The fixed striatal samples in 10% formalin from 4 rats in each group were dehydrated in successive alcohol solutions (methyl, ethyl, and absolute ethyl alcohol). Following dehydration, the samples were cleaned with xylene and embedded in paraffin wax for twenty-four hours at 56°C. Four-μm-thick slices of paraffin blocks were taken using a sledge microtome and mounted on slides, then deparaffinized and stained with hematoxylin and eosin stain (H&E) (Sigma Aldrich, St. Louis, MO, USA). Finally, light microscopy (Olympus BX41, Tokyo, Japan) was used to examine the stained striatal slides.

#### Transmission electron microscopy of the striatum

The isolated striatum was cut into approximately 1 mm cubes and fixed with precooled 2.5% glutaraldehyde. Then, the fixed samples were exposed to 1% osmium tetroxide. Following several washes, the samples were dehydrated with gradient ethanol, embedded in Epon, and finally polymerized. Regions of interest were identified, and ultrathin sections (40–70 nm) were spread on the grids. After the sections were stained with uranyl acetate and lead citrate, the examination was performed using transmission electron microscopy (TEM) (Jeol 1400 plus Tokyo, Japan) [26]. Four animals in each group were used for the TEM examination.

### Statistical analysis

All statistical analyses of the data were performed using Statistical Package for Social Sciences (SPSS), version 25 (IBM, Chicago, USA). The Shapiro-Wilk test was used to determine the data’s normality. The mean ± standard error of the mean (SEM) was used to express the results. A one-way analysis of variance (ANOVA) was used to assess the difference between groups and was followed by the Tukey post hoc test. Statistical significance was defined as a p-value of less than 0.05.

## Results

### Chemical characterization of the active extract

#### Phenolic and flavonoid contents

YA-C extract showed phenolic content of 8.1 ± 0.76 mg GAE/g extract and flavonoid content standardized at 1.66 ± 0.32 mg rutin/g extract. Thus, the YA-C extract was the most effective among the tested extracts against anti-inflammatory markers (TNF-R2 and NF-_*K*_B). Accordingly, it was subjected to detailed phytochemical and *in vivo* biological studies.

#### High-resolution LC-MS/MS metabolic profiling of *Y*. *aloifolia* fruit extract

**Fig 1:**
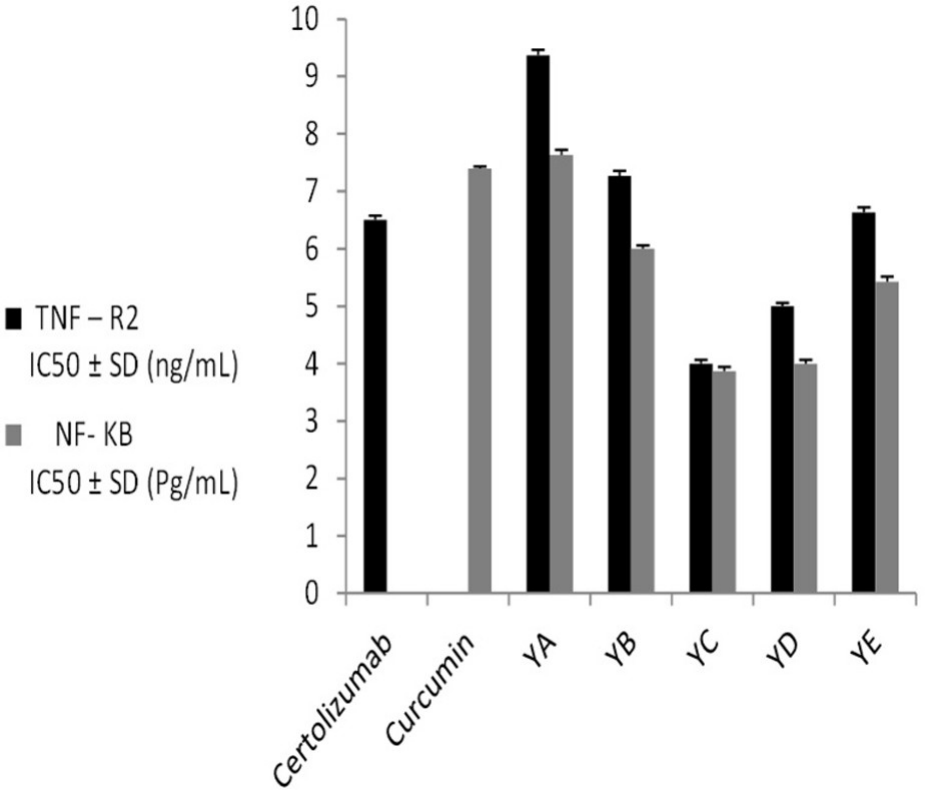
The effect of different fruit extracts on anti-inflammatory markers (TNF-R2 and NF-_*K*_ B).

**Fig 2:**
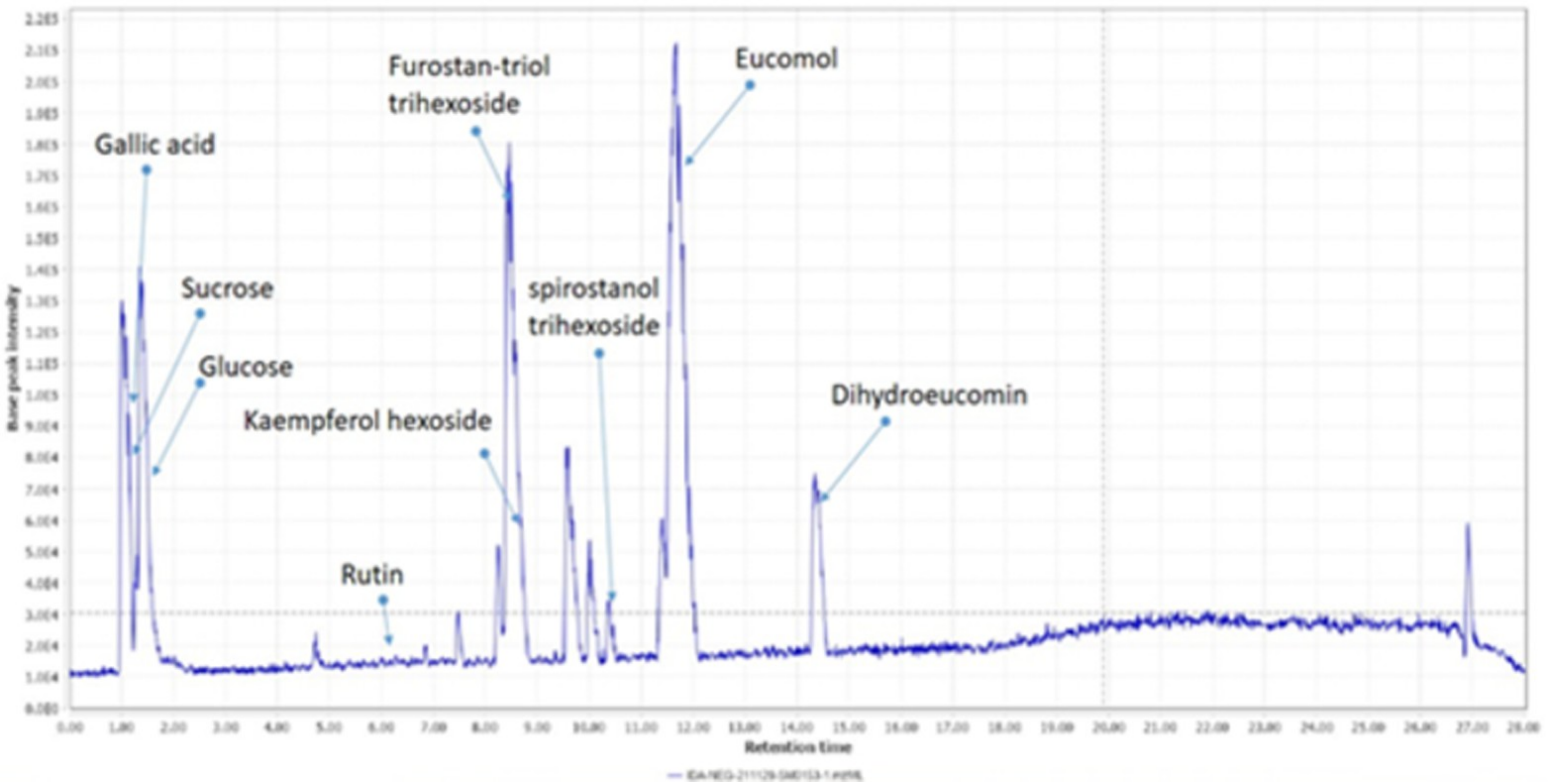
BPC of *Yucca aloifolia* fruit extract in negative ion mode.

A thorough tandem high-resolution mass analysis was carried out in both positive and negative modes to investigate the metabolites in the YA-C extract qualitatively. The main classes of the identified compounds were anthocyanins, sugars, phenolic acids, flavonoids, and saponins. The major compounds were tentatively identified by comparison of their exact masses to reported values in official databases such as the DNP, HMDB, and Phytohub, as well as those reported in the literature. The fragmentation pattern of the metabolites further confirmed the identification. Identified compounds are represented in (Table 1 and Figs 1 and 2).

**Table 1:** Major metabolites detected in *Yucca aloifolia* fruits via nano LC-HRMS/MS.

	Rt (min)	Identified compound	Mode	Molecular formula	Ion	Detected mass	MS/MS
1	1.17	Choline	P	C_5_H_14_NO	M+	104.1065	104, 58
2	1.25	Glucose	P	C_6_H_12_O_6_	M+Na	203.0529	203, 161
3	1.30		N	C_6_H_12_O_6_	M-H	179.0561	
4	1.28	Sucrose	N	C_12_H_22_O_11_	M-H	341.1093	
5	1.32		P		M+H	343.1232	343, 326, 258, 145
6	1.64	Gallic acid	N	C_7_H_6_O_5_	M-2H+Na	190.9500	
7	1.42	Delphinidin hexoside	P	C_21_H_21_O_12_	M	465.1032	465, 303
8	2.64	Cyanidin galactoside	P	C_21_H_21_O_11_	M	449.1080	449, 287
9	3.97	Cyanidin glucoside	P	C_21_H_21_O_11_	M	449.1083	449, 287
10	4.47	Pelargonidin hexoside	P	C_21_H_21_O_10_	M+	433.1140	433, 271
11	6.47	Rutin	P	C_27_H_30_O_16_	M+H	611.1608	611, 465, 356, 303
12	6.28		N	C_27_H_30_O_16_	M-H	609.1483	
13	6.81	Quercetin-O- hexoside	P		M+	465.1039	465, 303
14	8.46	Furostane-triol; trihexoside	P	C_45_H_76_O_19_	M+H-H2O	903.4955	903, 741, 579, 417, 273, 163
15	8.47		N	C_45_H_76_O_19_	M-H	919.4915	919, 757, 595,433
16	8.90	Kaempferol hexoside	N	C_22_H_23_O_10_	M-H	447.1294	447, 285, 165
17	10.28	Spirostan-3-ol; Glucopyranosyl- glucopyranosyl galactopyranoside]	N	C_45_H_74_O_18_	M-H	901.4791	901, 739, 323
18	10.34		P	C_45_H_74_O_18_	M+K	941.4507	941, 417, 273
19	10.38	Spirostan-3-ol; Glucopyranosyl glucopyranosyl glucopyranoside]	N	C_45_H_74_O_18_	M+HCOO	947.4873	901, 739, 323
20	11.67	Eucomol	N	C_17_H_16_O_6_	M-H	315.0875	315, 297, 282, 193, 178
21	14.36	Dihydroeucomin	N	C_17_H_16_O_5_	M-H	299.0916	299, 178, 150, 122
22	20.04	3,3’,5,5’-Tetrahydroxy-4-methoxystilbene	P	C_15_H_14_O_5_	M+ACN+H	316.1177	
23	23.27	Monopalmitin	P	C_19_H_38_O_4_	M+H	331.2850	331, 313, 239
24	26.10	Monostearin	P	C_21_H_42_O_4_	M+H	359.3159	359,(-18) 341, 285, 267

#### Sugars

Only two sugars were detected in YA-C fruit extract, identified as sucrose and glucose, as previously reported by [27]. Glucose was detected at m/z 203.0529 as its [M+ Na] adduct in the positive mode and its [M-H] ion at m/z 179.0561 in the negative mode. Shortly, it was followed by sucrose peaks representing [M-H] at 341.1093 and [M+H] at 343.1232.

#### Anthocyanins

This study thoroughly investigated anthocyanins due to their expected correlation to activity. The intense reddish-purple color of the fruit indicated its richness with anthocyanins which was further confirmed preliminary by the pH-dependent color test and later by LC-MS/MS. Three main aglycones were detected: cyanidin, delphinidin, and pelargonidin. These were mainly detected as their glycosides in the positive mode. A peak at R_t_ 1.4 min showed a molecular ion [M]^+^ at 465.1032 with an ms2 base peak at m/z 303 and was assigned to delphinidin hexoside.

The extracted ion chromatogram for cyanidin derivatives having a base peak at m/z 287 showed two peaks at m/z 449.1080 and 449.1083 at R_t_ 2.64 and 3.97, respectively. These were assigned to cyanidin galactoside followed by cyanidin glucoside due to the preferential earlier elution of galactosides [8]. Pelargonidin hexoside was detected in the positive mode at m/z 433.1140 and confirmed through the ms2 base peak at m/z 271 upon the loss of the hexose moiety.

#### Flavonoids

**Fig 3:**
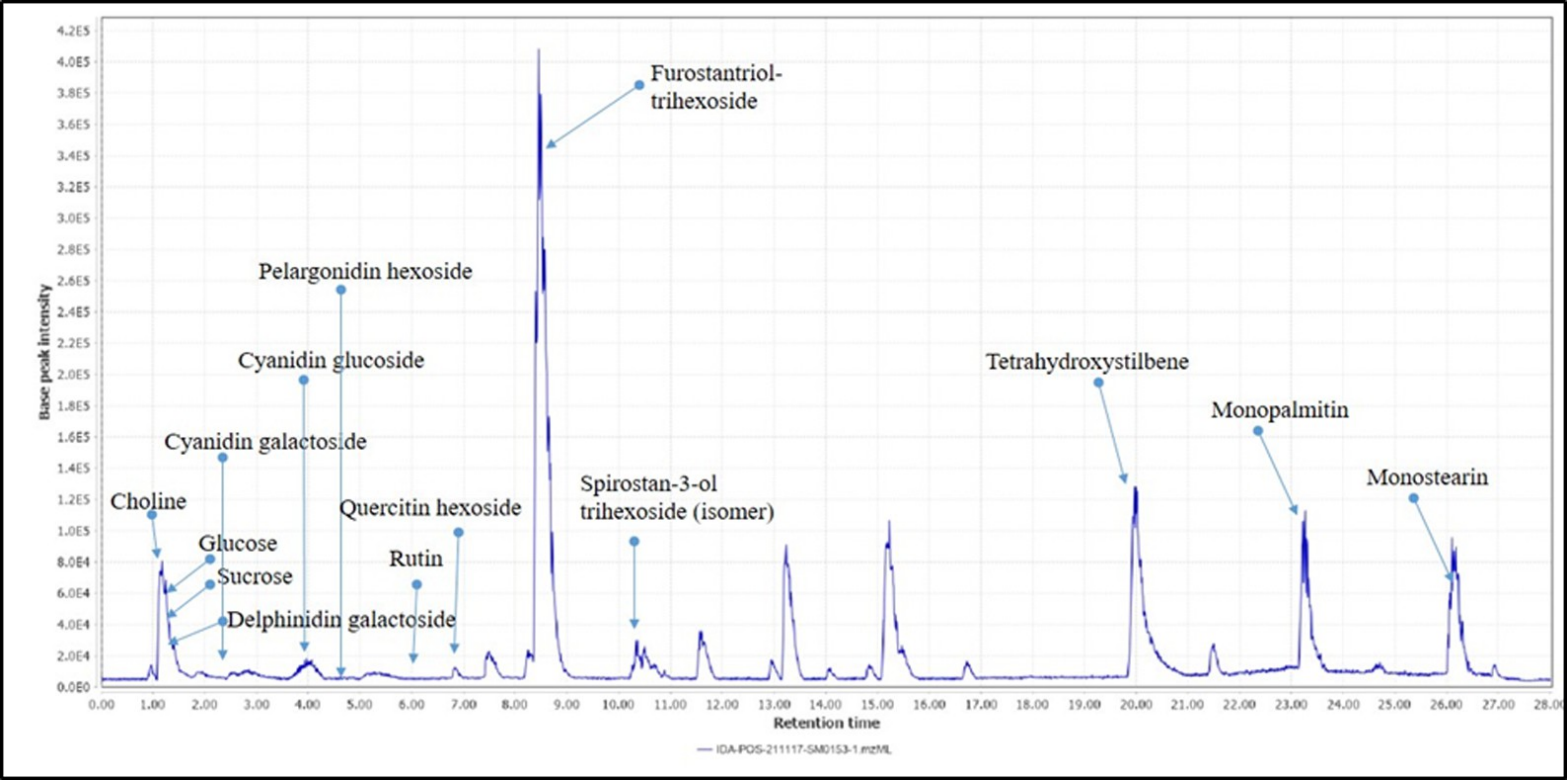
BPC of *Yucca aloifolia* fruit extract in positive ion mode.

Several flavonoid glycosides and aglycones were detected, mainly in the negative mode. Quercetin diglucoside (rutin) was detected in both modes and was confirmed compared to the standard sample using HPLC (Fig 3). It was observed as deprotonated and protonated molecular ions at m/z 609. 1483 and 611.1608. Fragmentation in the positive ion mode showed the base peak for the quercetin aglycone at m/z 303 and the intermediate peak at 465 due to the loss of the rhamnose moiety [M+H-146] [28]. Later, the monoglucoside quercetin hexoside was detected in the positive mode at its charged molecular ion at m/z 465.1028 at 6.81 min. A tetrahydroxyflavone hexoside was detected at 8.9 min as its deprotonated molecular ion at m/z 447.1286. It showed a fragment at 285 due to the loss of the hexose moiety. This peak was assumed to be a kaempferol derivative, not a luteolin one, due to the absence of ms2 fragments at m/z 175 and 107, characteristic of luteolin [29] and the presence of m/z 165 previously reported for kaempferol [30].

The peak at 11.67 min. was identified for the first time in *Yucca*, although previously reported in the family Agavaceae and detected at the same mass. The compound is known as eucomol, and its deprotonated ion was detected at m/z 315.0875 with an ms2 peak at m/z 297 due to the loss of a water moiety, followed by a characteristic fragment at low collision energy at m/z 193 due to cleavage at ring C, besides another at m/z 178 [8]. Its dihydro derivative, dihydro eucomin, was detected at m/z 299.096 at 14.36 min with the same fragment at m/z 178 [8].

#### Saponins

The genus *Yucca* is known to be rich in saponins. Leaves, stems, and roots were previously investigated, and their content of saponins was determined [8]. However, the fruit content of saponins still needs to be addressed. The LC-MS/MS analysis showed the presence of some saponins belonging to the furostanol and spirostanol groups. Generally, the plant contains both, although their ratio differs according to the collection season. Furostanol, the more active form, usually grows in the winter [31].

In both positive and negative modes, a furostanol trihexoside was detected at 8.46 min. In the positive mode, the [M+H-H_2_O]-ion was found at m/z 903.4955. Due to the open F-ring structure, the dehydrated molecular ion indicated a furostanol rather than a spirostanol [8, 32]. The ms2 fragments showed peaks at 741 and then at 579 and 417 due to the successive loss of three hexose sugar moieties, leading to the dehydrated aglycone at m/z 417. In the negative ion mode, the deprotonated molecular ion was detected at m/z 919.4915, and the ms2 fragments resulted from the stepwise loss of the three hexoses, giving peaks at 757, 595, and 433. Spirostanol trihexoside was detected in both positive and negative modes. Its deprotonated molecular ion was found at m/z 901.4790. The loss of a hexose led to a fragment at 739, followed by a fragment at 323 due to the loss of the aglycone of mass 416. In the positive mode, the ion [M+K]^+^ was at m/z 941.4507, and the base peak was at m/z 417, corresponding to spirostanol. Its isomer was detected later as its [M+HCOO]^-^ adduct with the same fragmentation pattern. The former isomer is believed to contain one of its hexose moieties, galactose.

#### Phenolic acids

Gallic acid was detected as its [M-2H+Na]^-^ at 190.9549, and its presence was further confirmed by comparison to the standard sample using HPLC (Fig 3).

#### Stilbenes

Tetrahydroxy Methoxystilbene was detected in the positive mode as its [M+CAN+H] at 20.04, and its exact mass was compared to that reported in databases.

#### Nitrogenous compounds

The essential biomolecule choline is known to enhance cognitive and neurological functions [33] and was detected in the positive mode at m/z 104.1064 and its ms2 fragments complied with those reported in the literature, mainly the fragment at m/z 58 [34].

#### Fatty acids

Monopalmitin, also known as 1-monopalmitate glycerol, was detected in the positive mode at 23.27 min at m/z 331.285. The ms2 fragments were identical to those previously reported [35], where ms2 at 313 corresponded to the loss of the water moiety, and ms2 at 239 was peculiar for the acyl chain of fatty acids [36, 37].

Another prominent peak was detected at 26.10 min. It was identified as monostearin at m/z 359.3159, and ms2 fragments were characteristic of fatty acid fragmentation, giving the dehydrated product ion at m/z 341 and the characteristic fragment at m/z 285 and 267 due to stearic acid and its dehydrated form [38]. The source of these fatty acids was expected to be the seeds enclosed in the fruit, as previously reported [9].

### *In vitro* biological study

#### Anti-inflammatory screening

**Fig 4:**
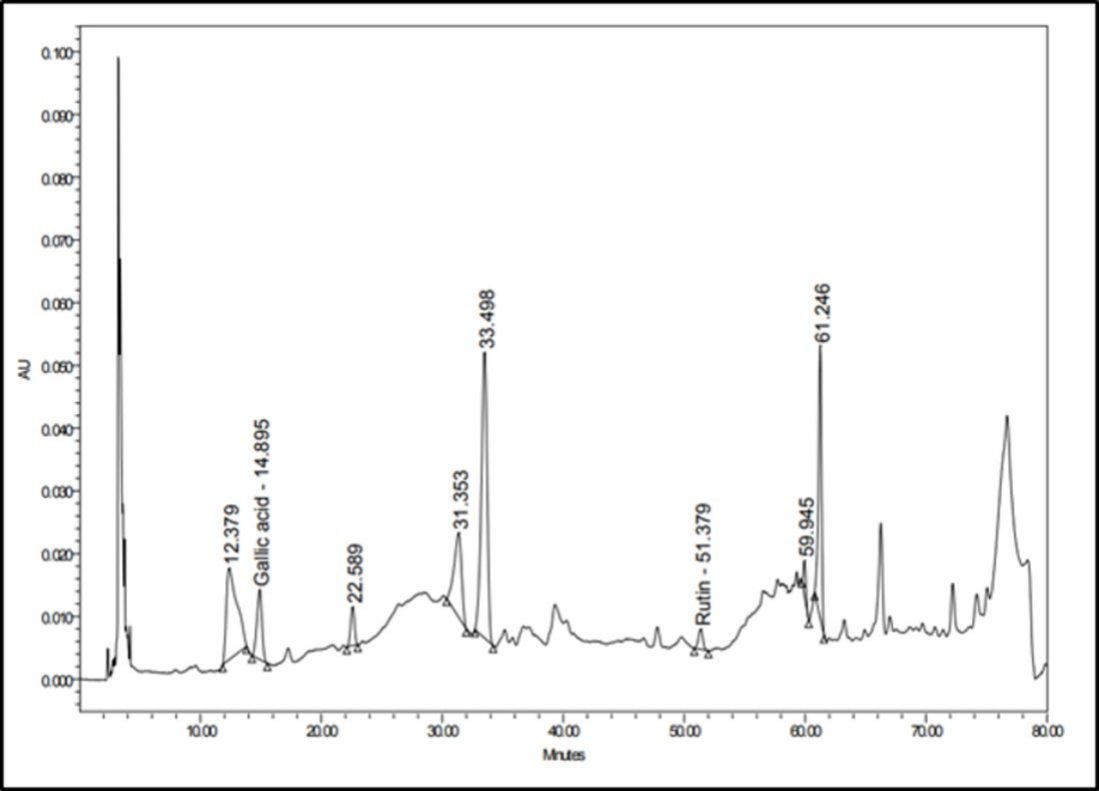
HPLC chromatogram of YC against rutin and gallic acid standards.

TNF-R2 and NF-_*K*_B were measured for all prepared extracts (YA-A to YA-E). The 50% inhibitory concentration (IC_50_) values were calculated from the concentration-inhibition response curve. Some prepared extracts showed a potent ability to bind with TNF-R2 (TNF-Receptors). Furthermore, the acidified 50% ethanol extract (YA-C) demonstrated the highest binding affinity with IC_50_ 4 ± 0.06 ng/mL, which was lower than the positive standard certolizumab with IC_50_ 6.5 ± 0.07 ng/mL (Fig 4). Moreover, the inhibition of NF-_*K*_B by extract YA-C showed IC_50_ of 3.87 ± 0.07 Pg/mL, which was lower than that of curcumin standard (7.4 ± 0.03 Pg/mL) (Fig 3).

### *In vivo* biological study

#### Effect of Yucca extract (YA-C) on body weight change

Rats treated with low and high doses of *Yucca* extract (YA-C) showed a non-significant change in weight, and both groups gained weight as the control group (*P* > 0.05; Table 2).

**Table 2:** Effect of different doses of *Yucca* extract (YC) on weight, neurobehavioral and locomotor tests, renal function, liver enzymes, and dopamine.

Parameter	Normal control (NC)	Low-dose *Yucca* (YC) extract (50mg/kg)	High-dose *Yucca* (YC) extract (100mg/kg)	P-value
**Weight change (g)**	32.00 ± 5.57	36.88± 5.67	29.63±5.82	0.661
**Rota rod test** Mean latency to fall	96.00 ± 1.48	99.94 ± 1.10	99.91 ± 2.34	0.199
**Open field** Latency to move in (Seconds)	4.13 ± 0.30	3.88 ± 0.30	3.75 ± 0.25	0.636
Number of crossed squares	27.20 ± 0.38	27.13 ± 0.79	28.00 ± 0.63	0.614
Latency to rear (Seconds)	12.63 ± 0.68	12.63 ± 0.65	11.63± 0.60	0.461
Number of rears	11.13 ± 0.72	11.13 ± 0.74	11.38 ± 1.07	0.972
**Forced stress swim test** Swimming time (Seconds)	122.75 ± 1.77	125.38 ± 2.05	128.75± 2.48	0.159
Climbing time (Seconds)	91.75 ± 3.51	92.25 ± 3.47	95.38 ± 2.67	0.623
Immobility time (Seconds)	85.13 ± 1.85	82.38 ± 1.69	78.50 ± 2.67	0.107
**Renal function** Urea (mg/dl)	50 ± 1.82	53.88 ±2.07	55.50 ± 2.67	0.344
Creatinine (mg/dl)	0.59 ± 0.02	0.56± 0.04	0.57 ± 0.02	0.680
**Liver enzymes** SGOT (U/L)	197.25 ± 2.94	198.88 ± 2.98	201.63 ± 4.48	0.682
SGPT (U/L)	92.25 ± 4.06	84.13 ± 4.46	80.75 ±4.50	0.182
**Dopamine** (μg/mg protein)	13.39 ± 0.32	13.32. ± 0.39	13.30 ±0.29	0.980

Data are represented as the mean ± SEM of eight animals in each group.

#### Effect of *Yucca* extract (YA-C) on rats’ behavior

Compared to the control group, the administration of YA-C extract with both doses did not demonstrate a significant change in the time spent on the rod (*P* > 0.05). Concerning the open field apparatus, the exposure to YA-C did not result in any apparent delay in the time to move, the number of crossed squares, or the rearing of rats (*P* > 0.05). These findings rule out a negative impact of YA-C extract on rats’ motor and memory abilities. Furthermore, they performed similarly to normal rats in the forced swim test regarding swimming, climbing, and immobility time, indicating a nondepressive effect of the tested doses (*P* > 0.05; Table 2).

#### Effect of *Yucca* extract on renal function and liver enzymes

The two doses of YA-C extract demonstrated non-significant changes in SGOT, SGPT, urea, and creatinine compared with the normal control group (*P* > 0.05; Table 2). These findings confirmed the hepatic and renal safety of these doses.

#### Effect of *Yucca* extract on striatal dopamine content

The levels of striatal DA were not significantly different between the control and YA-C extract-treated groups (*P* > 0.05; Table 2). As a result, the given doses had a non-degenerative effect on nigrostriatal dopaminergic neurons, as confirmed by striatal histopathological examination.

#### Effect of *Yucca* extract on striatal histology

**Fig 5:**
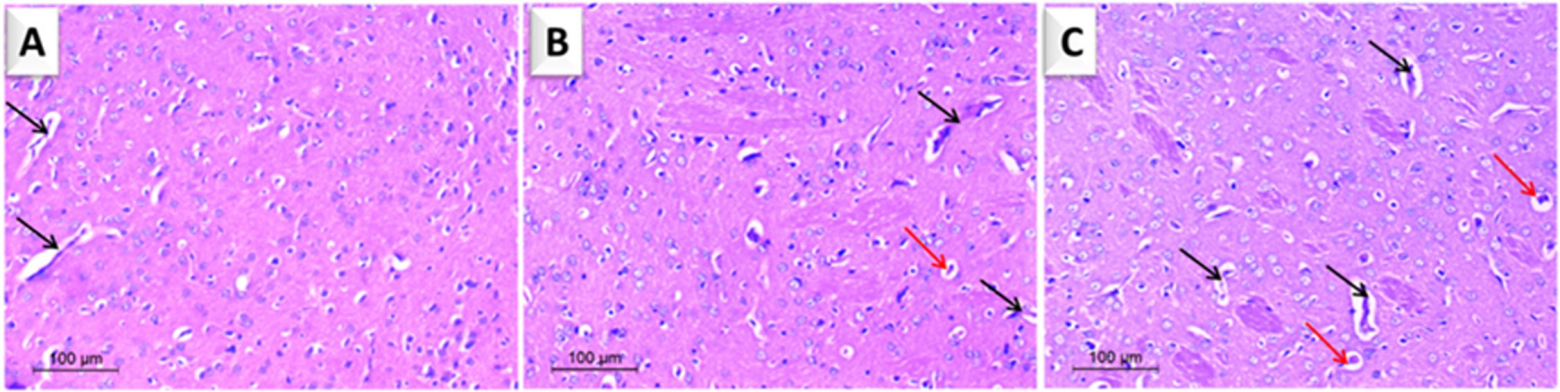
Photomicrographs of rat striatum (H & E, 100). **(A)** Normal neurons with some blood vessels as a control (black arrows). (**B**) Low dose Yucca (50 mg/kg) and (**C**) high dose yucca (100 mg/kg): perineuronal vacuolation (red arrows), blood vessel (black arrows).

By LM examination of the striatal tissue for both dosages compared to the control section, no features of injury or toxicity were observed. Moreover, a non-significant increase in the number of blood vessels was found, indicating a possible vascularizing effect of the extract (Fig 5A–5C).

### Mechanistic study

#### Effect of YA-C extract on body weight change of rotenone-treated rats

Four weeks of PD induction with rotenone resulted in an apparent decrease in body weight compared with their initial weight. Co-treatment of rats inhibited the loss of weight with YA-C extract. At the same time, the treated rats’ weight was statistically indistinguishable from the non-diseased rats (*P* < 0.001; Table 3).

**Table 3:** Effects of rotenone and its co-treatment with *Yucca* extract on rats’ weight and behavior.

	Control	Rotenone	Rotenone+ low dose *Yucca*	Rotenone + high dose of *Yucca*	P-value
**Weight change (g)**	26.50 ± 3.00	-3.6 ±1.89*	11.63 ±4.59#	15.38 ±5.91#	<0.001
**Rota rod test** (Mean latency to fall)	95.75 ±1.52	29.58 ±1.1*	65.25 ±1.79*#	88.54 ±1.19*#≡	<0.001
**Open field** Latency to move in (seconds)	3.75 ±0.25	45.63 ±2.18*	29.25 ±4.51*#	13.88 ±0.88*#≡	<0.001
Number of crossed squares	27.25 ±0.56	12.25 ±0.70*	17.13 ±0.35*#	23.38 ±0.53*#≡	<0.001
Latency to rear (seconds)	12.50 ±0.71	35.63 ±2.37*	22.75 ±1.99*#	16.25 ±0.45#≡	<0.001
Number of rears	11.00 ±0.71	3.13 ±0.30*	6.75 ±0.25*#	10.25 ±0.45#≡	<0.001
**Forced stress swim test** Swimming time (seconds)	122.50 ±1.72	78.13 ±1.38*	94.25 ±1.94*#	112.00 ±2.95*#≡	<0.001
Climbing time (seconds)	90.75 ±3.50	76.38 ±0.84*	89.50 ±2.49#	93.75 ±2.58#	<0.001
Immobility time (seconds)	86.75 ±1.93	145.50 ±1.32*	116.25 ±2.24*#	94.25 ±1.25*#≡	<0.001

Data are represented as the mean ± SEM of eight animals in each group.

* Significantly differs (P < 0.05) compared to the control group;

# significantly differs (P < 0.05) compared to rotenone alone-treated group;

≡ significantly differs (P < 0.05) compared to rotenone + Yucca (50 mg/kg) treated group.

#### Effect of *Yucca* extract on rotarod performance of rotenone-treated rats

The rotenone group spent significantly less time on the rod than the control group. The co-administration of YA-C extract with rotenone resulted in a significant increase in the rotarod time compared to the rotenone group. The latency to fall from the rod was significantly increased when the *Yucca* dose was doubled (*P* < 0.001; Table 3).

#### Effect of *Yucca* extract on the open-filed performance of rotenone-treated rats

The motor activity of rats exposed to rotenone showed a significant delay in moving to the rear and a significant reduction in the number of crossed squares and rears compared to controls. Treatment of rats exposed to rotenone with different doses of YA-C extracts significantly increased the mobility time and the number of crossed squares and rears compared to the rotenone-treated rats. Compared to the enhanced effect of a lower dose on motor activity, doubling the YA-C dose resulted in a more substantial improvement in motor function in rats (*P* < 0.001).

#### Effect of *Yucca* extract on forced swim test of rotenone-treated rats

Rotenone-treated rats exhibited a significant delay in swimming and climbing times and a prolongation in immobilization compared with normal and YA-C extract-treated rats. These results indicated depressive-like behavior of rotenone, which was improved by treatment of rotenone-exposed rats with different doses of YA-C extract (*P* < 0.001; Table 3).

#### Effect of *Yucca* extract on striatal oxidative and anti-oxidative stress biomarkers (MDA, NO, GSH, and SOD) of rotenone-treated rats

**Fig 6:**
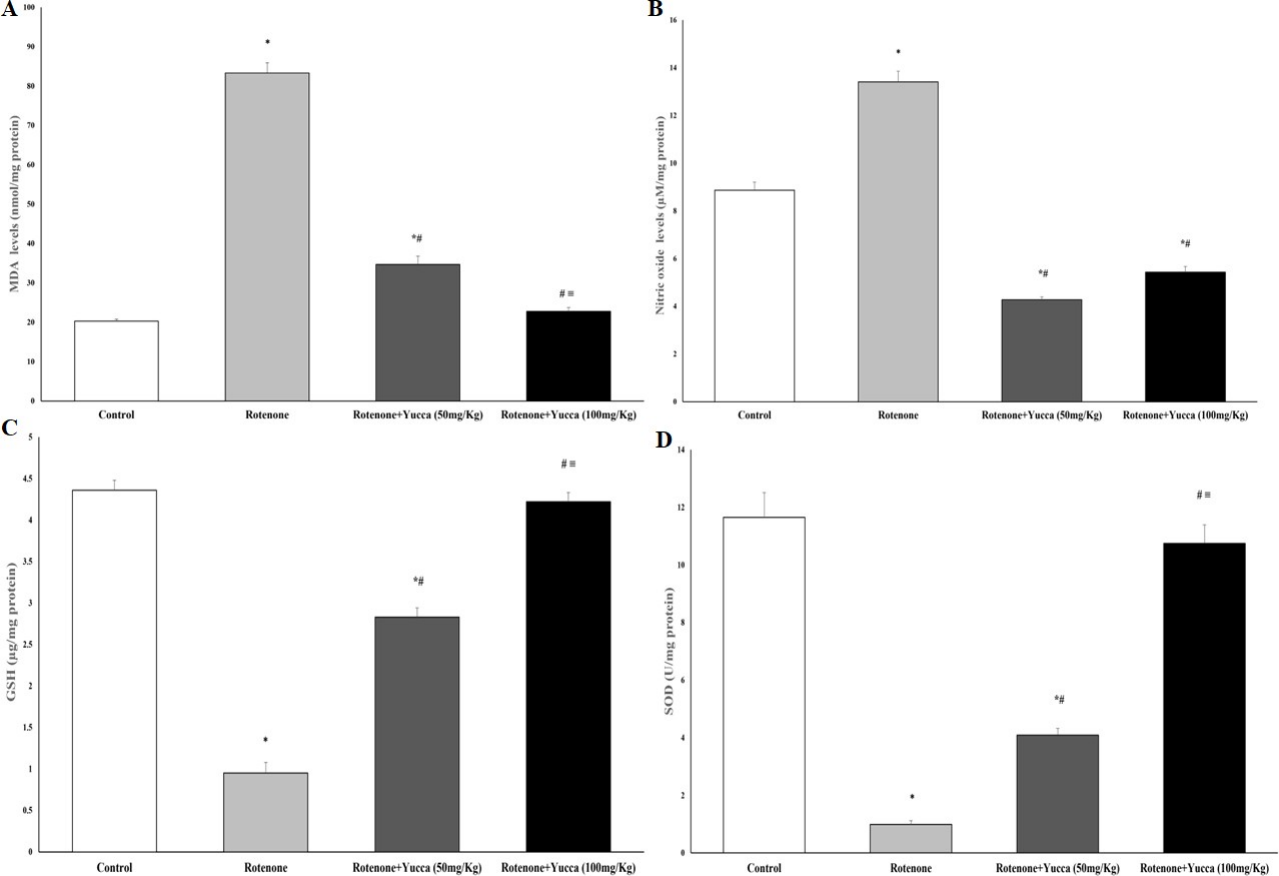
Effect of rotenone and its cotreatment with yucca extract on oxidative and antioxidative stress markers. **A, B**: cotreatment of yucca extract with rotenone decreased MDA and nitric oxide. **C, D**: increased GSH and SOD in rats’ striatum. Data are represented as the mean ± SEM of eight animals in each group. (*) Significantly differs (*P* < 0.05) compared to control group; (#) significantly differs (*P* < 0.05) compared to rotenone alone treated group; (≡) significantly differs (*P* < 0.05) compared to rotenone + yucca (50mg/kg) treated group.

The striatal levels of MDA and NO in the rotenone group were significantly increased. At the same time, GSH and SOD levels were significantly decreased compared to the control and YA-C extract-treated groups (*P* < 0.001). In comparison to the rotenone group, YA-C extract dose-dependently reduced oxidative stress and increased GSH and SOD (*P* < 0.05, Fig 6).

#### Effect of *Yucca* extract on striatal dopamine content of rotenone-treated rats

**Fig 7:**
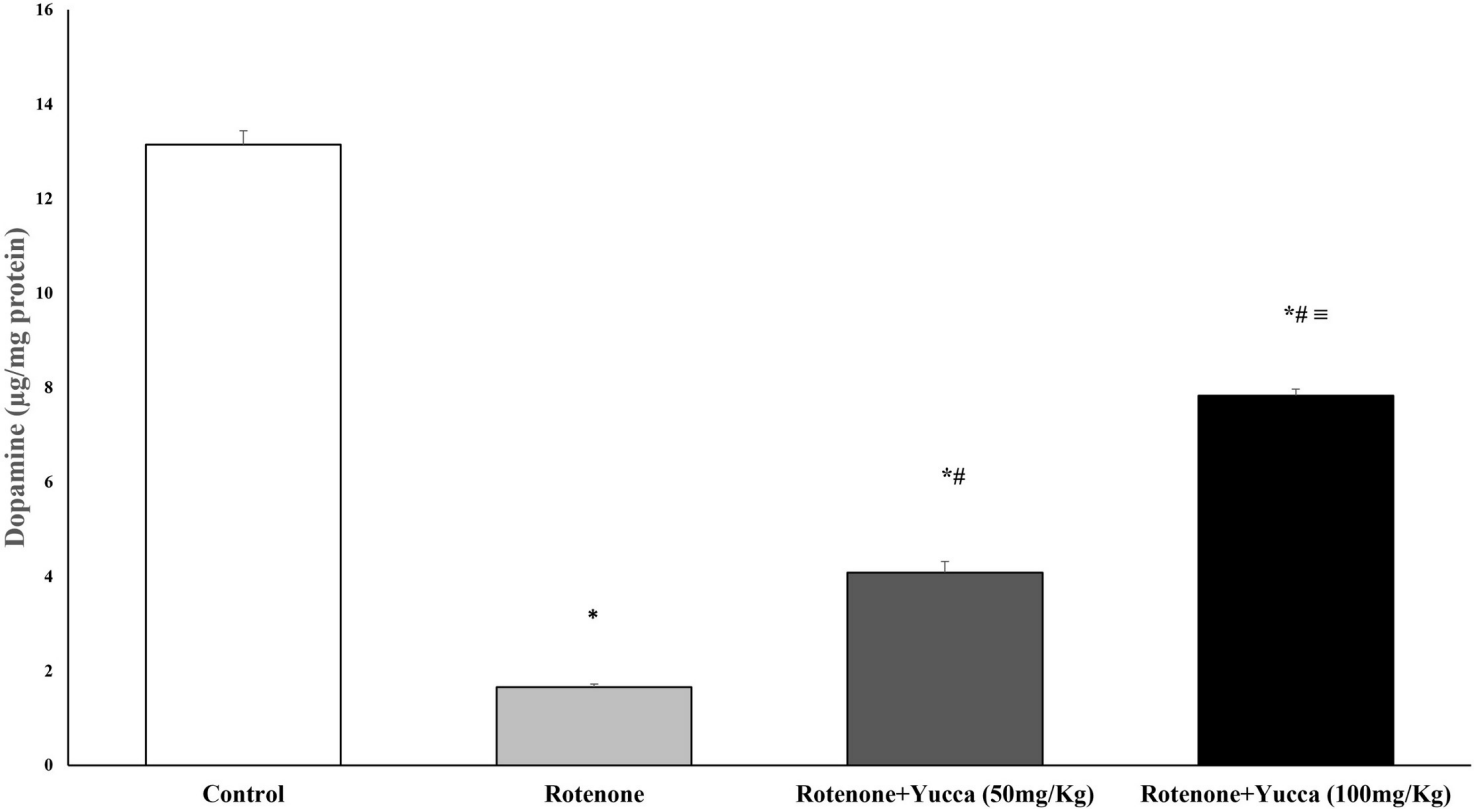
Effect of rotenone and its cotreatment with yucca extract on striatal Dopamine (μg/mg protein). Data are represented as the mean ± SEM of eight animals in each group. (*) Significantly differs (*P*< 0.05) compared to control group; (#) significantly differs (*P*< 0.05) compared to rotenone alone treated group; (≡) significantly differs (*P*< 0.05) compared to rotenone + yucca (50mg/kg) treated group.

Compared to the control group, the administration of rotenone for four weeks resulted in a substantial drop in striatal DA content. Treatment of rats with YA-C extract resulted in a significant increase in DA levels compared to the rotenone group, which was nearly doubled with a higher dose of YA-C extract (*P* < 0.001, Fig 7).

#### Effect of *Yucca* extract on striatal p-AMPK, Wnt3a, and β-catenin of rotenone-treated rats

**Fig 8:**
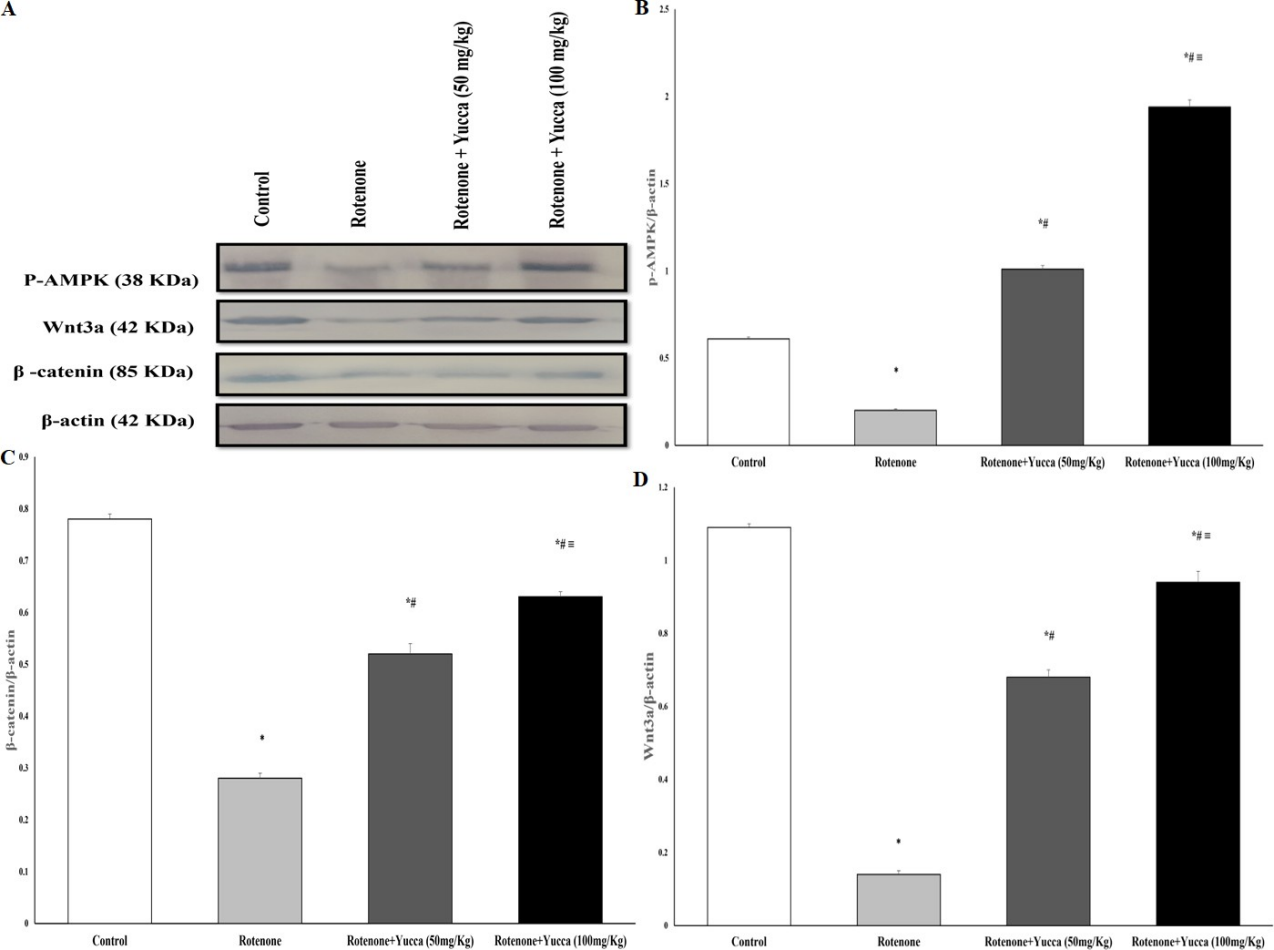
(**A**) The protein expression of p-AMPK, β-catenin and Wnt3a determined by western blot analysis. (**B-D**): the relative band intensities of p-AMPK, β-catenin and Wnt3a adjusted to the expression of *β*-actin. Co-treatment of yucca extract with rotenone increased protein expression of (B) p-AMPK, (**C**) β-catenin (**D**) Wnt3a. Data are represented as the mean ± SEM of eight animals in each group. (*) Significantly differs (*P*< 0.05) compared to control group; (#) significantly differs (*P*< 0.05) compared to rotenone alone treated group; (≡) significantly differs (*P*< 0.05) compared to rotenone + yucca (50mg/kg) treated group.

The protein expression of p-AMPK, Wnt3a, and β- catenin was downregulated in the rotenone-treated group to significant levels. In a dose-dependent manner, treatment with YA-C extract (50 and 100 mg/kg) decreased the effect of rotenone on these striatal parameters significantly and upregulated their expression (*P* < 0.001, Figs 8 and S1).

### Histopathological examination of striatal neurons

**Fig 9:**
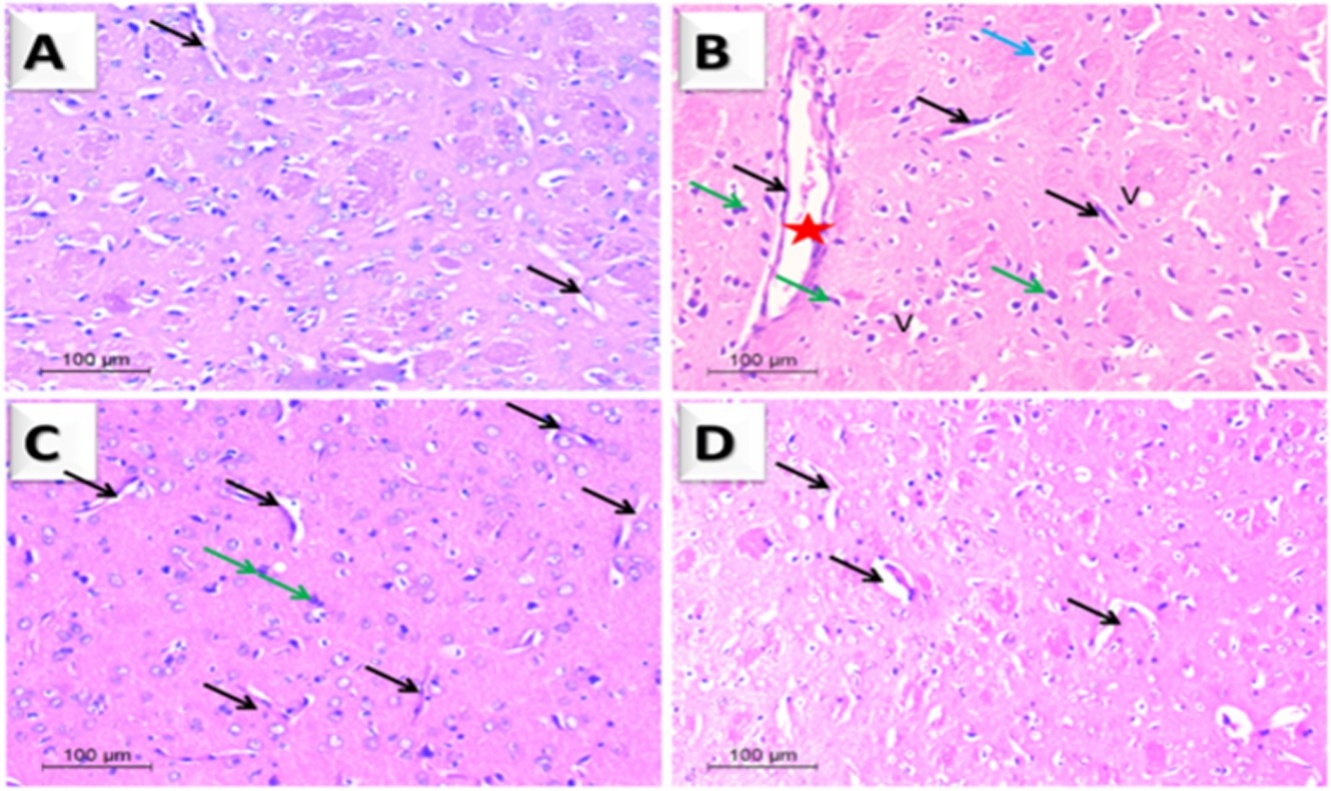
Photomicrographs of rat striatum (H & E, 100). (**A**) normal neurons with some blood vessels as a control (black arrows). (**B**) Rotenone (positive control group): epineuronal vacuolation (v), dilated blood vessel congested (star), and vacuolated neuropil (blue arrow) pyknotic neuclei neuron (green arrows). (**C**) Rotenone + Yucca 50mg/kg: pyknotic (green arrows) and vascularization remain (black arrow). (**D**) Rotenone + yucca 100 mg/kg: nearly normal tissue with a small number of blood vessels (black arrow).

The control group’s striatal sections revealed a typical architecture with neurons sustaining their structure and regular cellularity containing visible nuclei. Neurons were grouped in tidy rows with abundant cytoplasm and spherical basophilic nuclei (Fig 9A). H&E-stained sections in the rotenone-treated group indicated severe neurodegenerative changes, such as extensively dark pyknotic areas, apoptotic nuclei, chromatin condensation in neurons, vacuolation, and congested vascular channels. Moreover, congested capillaries with red blood cells were observed. All of the above symptoms reflect changes associated with Parkinson’s pathophysiology (Fig 9B). These pathological changes were alleviated dose-dependently by treatment with YA-C. Sections of rats treated with a 50 mg/kg dose showed moderate improvement except for occasional pyknosis and perivascular vacuolation. Meanwhile, sections of 100 mg/kg dose revealed virtually normal striatal neuronal cells (Fig 9C and 9D).

**Fig 10:**
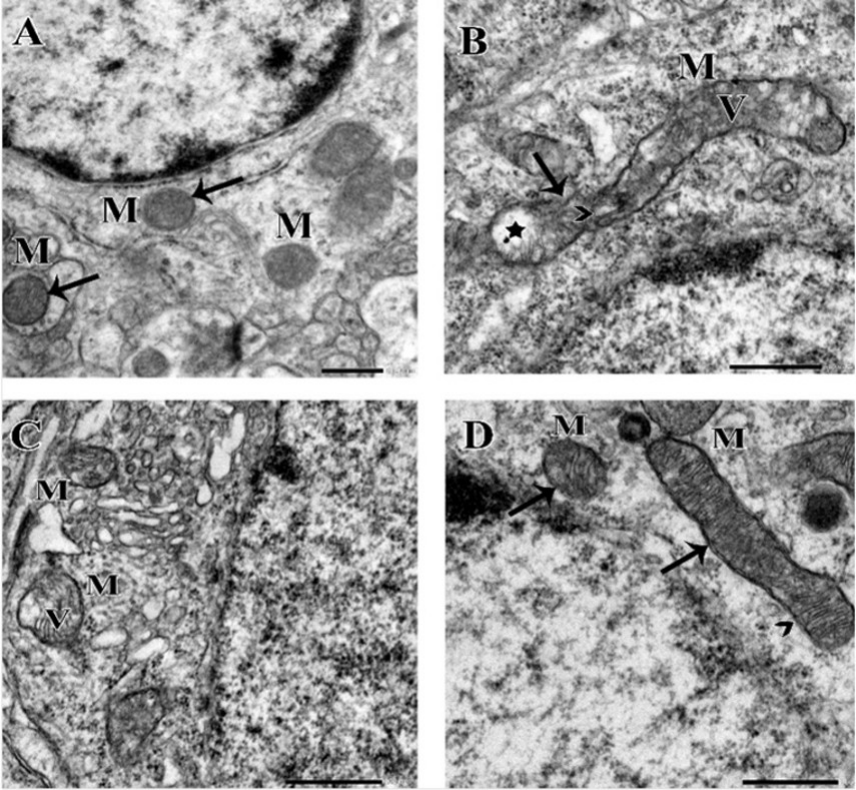
Electron microscopic pictures showing morphological changes in mitochondria of the striatal neurons of rats treated with different doses of yucca alone or with rotenone. **(A)** The control group shows normal mitochondria (M) with their double membranes (arrows), regular cristae and healthy matrix. **(B)** A micrograph of the rotenone group showing a disintegrated mitochondrion (M), with discontinuity of its membranes (arrow). The cristae are distorted (arrowhead) and the matrix shows hypointensity (*) and vacuolization (V). **(C)** A micrograph of the rotenone + low dose of yucca group shows mild improvement. The mitochondrial membranes are less disrupted, but the matrix still shows vacuoles (V). **(D)** A micrograph of the rotenone + high dose of yucca group showing a marked improvement of the mitochondria (M), with continuous membranes (arrows), regular lamellar cristae (arrowhead), and apparently normal matrix.

EM examination of the striatum revealed normal mitochondria in the control group that disintegrated with distorted cristae in the rotenone group. On the other hand, treated rat striata with YA-C extract revealed amelioration of distorted mitochondrial features and more improvement with the higher YA-C dose (Fig 10A–10D).

## Discussion

The current phytochemical study revealed that *Y*. *aloifolia* fruit extract contained many active metabolites that contributed to brain health and were proven to protect against rotenone-induced PD. The tentative anthocyanins such as cyanidin, delphinidin, and pelargonidin derivatives had high neuroprotective, inhibition of oxidative stress, and oligomer-induced neuronal destabilization and suppression of DA oxidation in CNS disorders [39]. In addition, they showed the presence of antioxidant phenolic acid derivatives and anti-inflammatory steroidal saponin glycosides [40].

Additionally, the *Yucca* extract was rich in gallic acid, which, through its antioxidant activity, could improve peripheral nerve degeneration upon induced nerve injury [41]. Moreover, the flavonol quercetin and its glycosides demonstrated anti-oxidative, anticancer, and neuroprotective effects in Alzheimer’s and Parkinson’s disease through autophagy enhancement and other mechanisms [39, 42]. Besides, kaempferol lowers lipid peroxidation and protects from brain damage caused by reactive oxygen species (ROS) [39].

Additionally, the presence of choline, one of the extract’s primary compounds, explained its excellent potential for ameliorating the symptoms of neurodegenerative diseases. The homoisoflavonoid eucomol, identified for the first time in *Yucca*, was expected to contribute to the activity. Recent studies suggested that homoisoflavonoids acted as selective MAO-B inhibitors, which were used with L-DOPA or dopamine agonists in symptomatic PD [43].

Following the in-vivo safety study of *Y*. *aloifolia* extract (YA-C), experimental PD was modulated in rats by subcutaneous rotenone, and a lipophilic natural pesticide readily diffused through the blood-brain barrier [44]. The rotenone optimally produced pathological and clinical symptoms relatable to PD, as demonstrated by the significant drop in striatal DA and anti-oxidative stress biomarkers (GSH and SOD) and the neurodegenerative changes in the H&E-stained sections of the rotenone-treated group when compared to the normal rats.

The mean latency evidenced motor abnormalities associated with PD to fall time, latency to move, several crossed squares, latency to the rear, and several rears performed in rotarod and open field testing, all of which showed poor performance of the rotenone group. In contrast, depressive behavior associated with PD was manifested in the rotenone group as decreased swimming and climbing time and increased immobility time through the forced swim test, confirmed by previous literature reports [2, 45, 46].

The *Yucca* fruit YC extract demonstrated high anti-inflammatory activity (*in vitro*) based on the changes in the levels of anti-inflammatory markers (TNF-R2 and NF-_*K*_B). In contrast, in the *in vivo* study, a dose-dependent effect was achieved with YA-C fruit extract [7], as detected by improved striatal DA content, increased anti-oxidative stress capacity, and enhanced rats’ performance in the rotarod, open field, and forced swimming tests that confirmed a neuroprotective and antioxidant effect of *Yucca* fruit extract.

Evidence from previous studies indicated that impairment of mitochondrial complex I dynamics strongly contributed to dopaminergic cell death in PD [47], and *in vitro* treatment of dopaminergic cell lines with anthocyanins-rich extracts partially reversed rotenone-induced mitochondrial dysfunction, which was in line with our electron microscope findings and suggested a protective effect of *Yucca* extract on mitochondria.

In this study, the Wnt/beta-catenin/AMPK pathway was studied as a possible mechanism implicated in the pathogenesis of PD and a possible target of anthocyanins. Western blot analysis showed that rotenone significantly decreased protein expression in rats, and YA-C treatment partially restored the protein levels. These findings supported the previous research suggesting the possible modulation of the Wnt/β-catenin/ AMPK pathway by anthocyanin-rich plants [6, 7].

## Conclusion

The current study illustrated through the measurement of different comprehensive neurological parameters the significant effect of the acidified 50% ethanol extract (YA-C) of the edible yucca fruit compared to well-known standards and suggested a promising new therapeutic target (Wnt/Beta-catenin/AMPK pathway) for PD. The fruit can be a vehicle for a myriad of active constituents, and all combine to orchestrate the neuroprotective activity of the fruit.

## Supporting information

S1 Fig Western blot analysis determined the protein expression of p-AMPK, β-catenin, and Wnt3a. (PDF)
