# Supplementary figures and images for: HR LC-MS/MS metabolomic profiling of Yucca aloifolia fruit and the potential neuroprotective effect on rotenone-induced Parkinson’s disease in rats
Source: PLoS One. 2023 Feb 28;18(2):e0282246. doi: 10.1371/journal.pone.0282246 (PMC9974117; doi:10.1371/journal.pone.0282246)

**Repeat 1**

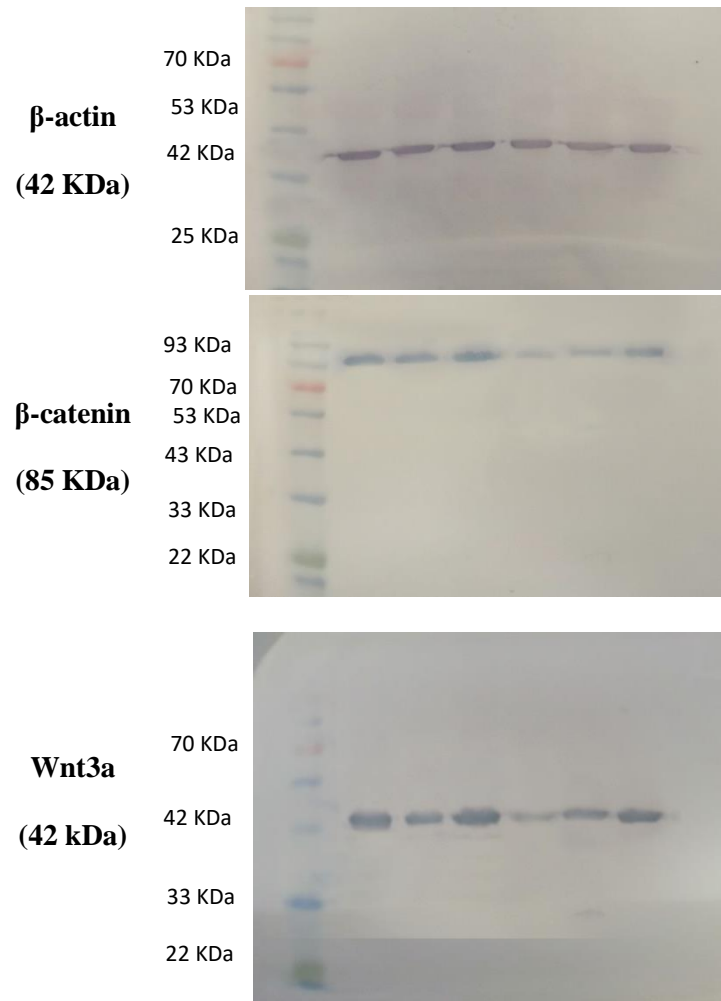

**Repeat 2**

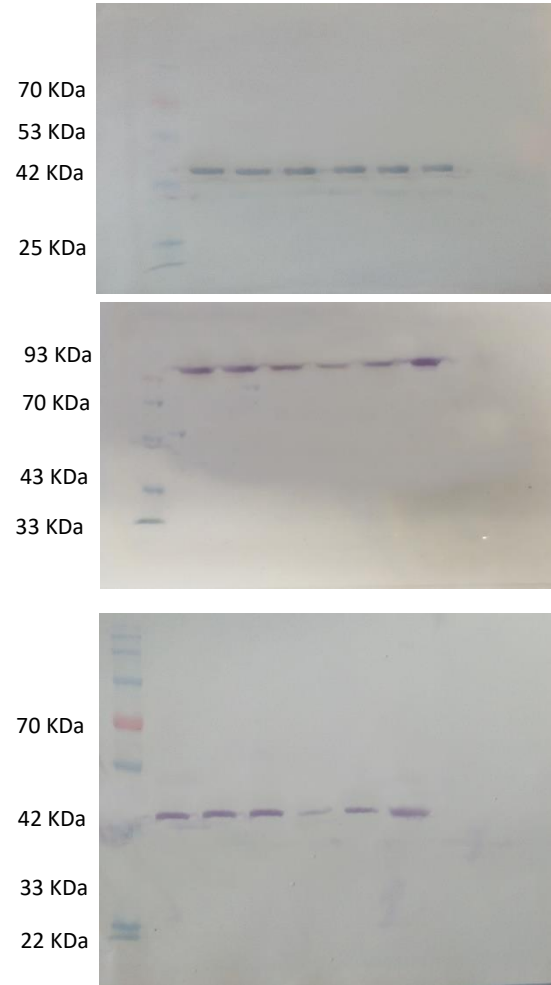

**Repeat 3**

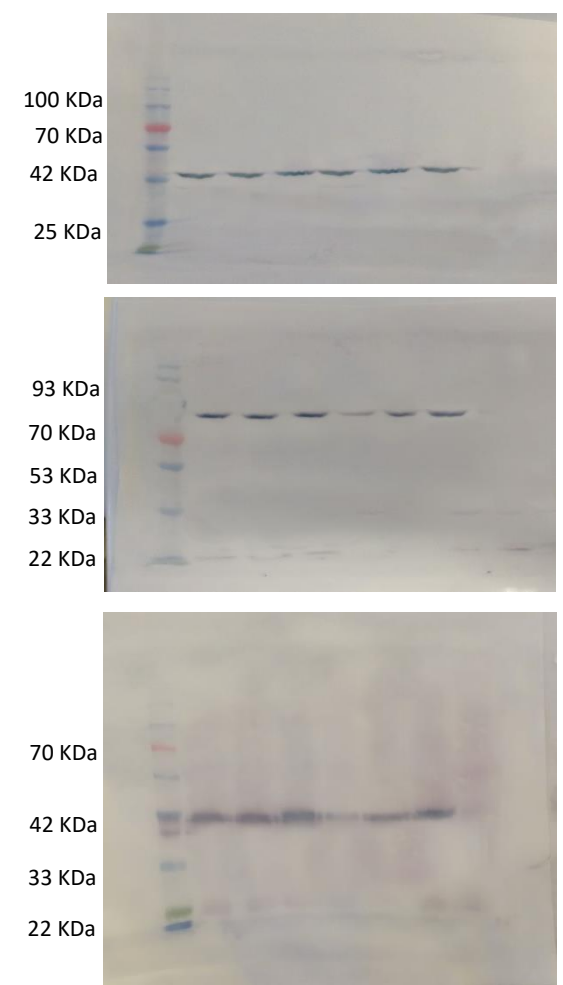

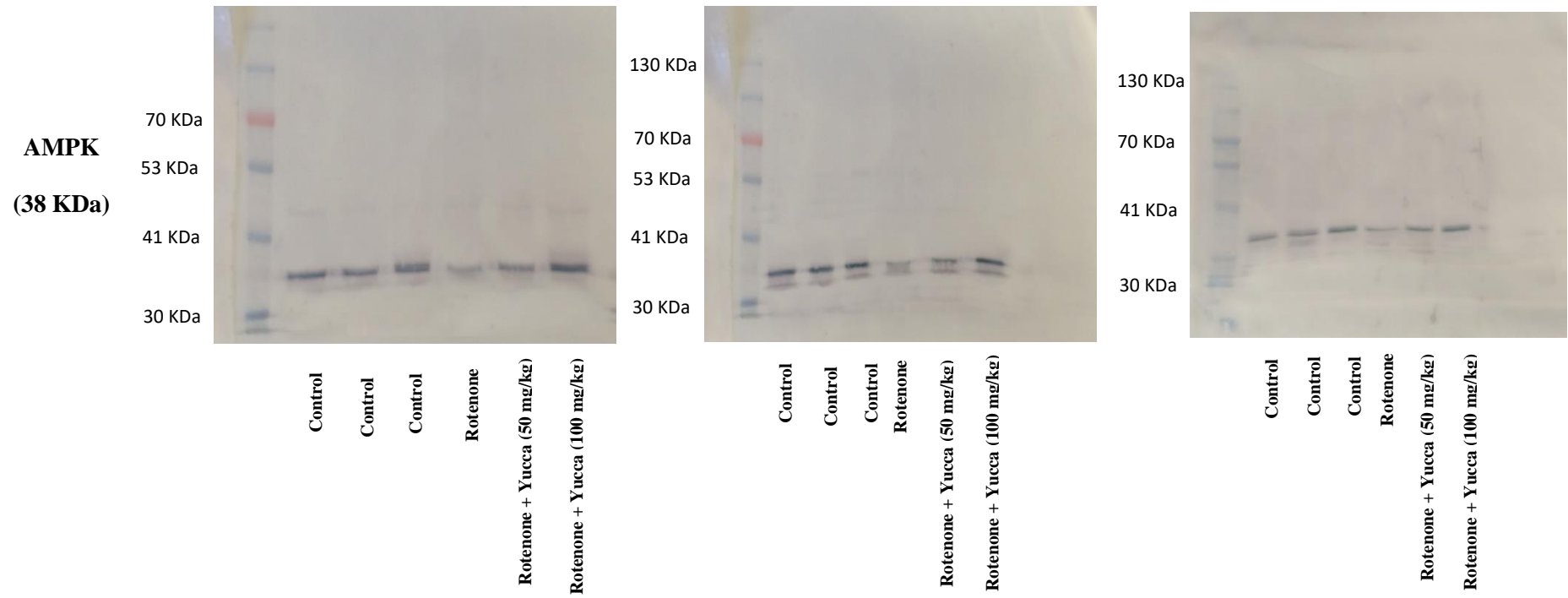

**Fig. 1. Original uncropped western blot for three repeats of each protein.**

Supplement: S1 Fig — (PDF) [file pone.0282246.s001.pdf]
